# Identifying Endogenous Cellular Proteins Destabilizing the Propagation of Swi1 Prion upon Overproduction

**DOI:** 10.3390/v14071366

**Published:** 2022-06-23

**Authors:** Zhiqiang Du, Brandon Cho, Liming Li

**Affiliations:** Department of Biochemistry and Molecular Genetics, Feinberg School of Medicine, Northwestern University, Chicago, IL 60611, USA; brandoncho2020@u.northwestern.edu

**Keywords:** protein aggregation, prion propagation, prion inhibitors, Swi1, [*SWI*^+^], SWI/SNF, yeast, *Saccharomyces cerevisiae*

## Abstract

(1) Background: Numerous prions exist in the budding yeast, including [*SWI*^+^], the prion form of Swi1—a subunit of the chromatin-remodeling complex SWI/SNF. Despite decades of research, the molecular mechanisms underlying prion initiation and propagation are not fully understood. In this study, we aimed to identify endogenous cellular proteins that destabilize [*SWI*^+^]. (2) Methods: We screened the MoBY-ORF 2.0 library for proteins that destabilize [*SWI*^+^] upon overproduction. We further explored the effects of the identified candidates against other yeast prions and analyzed their potential prion-curing mechanisms. (3) Results: Eighty-two [*SWI*^+^] suppressors were identified, and their effects were shown to be [*SWI*^+^]-specific. Interestingly, a few documented [*SWI*^+^] suppressors were not among the identified hits. Further experiments indicate that, for some of these [*SWI*^+^] suppressors, their overproduction, and thus their prion-curing activities, are regulated by environmental conditions. Bioinformatics analyses show that our identified [*SWI*^+^] suppressors are involved in diverse biological functions, with gene ontology term enrichments specifically for transcriptional regulation and translation. Competition for Swi1 monomers between [*SWI*^+^] and Swi1 interactors, including the SWI/SNF complex, is a potential prion-curing mechanism. (4) Conclusions: We identified a number of [*SWI*^+^]-specific suppressors that highlight unique features of [*SWI*^+^] in maintaining its self-perpetuating conformations.

## 1. Introduction

Prions are self-propagating protein conformations, initially identified as infectious agents causing mammalian transmissible spongiform encephalopathies or prion diseases [1]. This prion concept of protein-based infectivity has now been implicated in several human amyloid-based diseases, such as Alzheimer’s disease, Parkinson’s disease, amyotrophic lateral sclerosis, and type 2 (late-onset) diabetes [2,3,4,5]. Interestingly, multiple prion-forming proteins have been also discovered in *Saccharomyces cerevisiae*, which are transmitted as altered protein conformations that are linked to changes in phenotypes and thus referred to as yeast prions (see recent reviews of [6,7,8,9,10,11,12,13]). Studies from yeast prions have provided valuable information regarding the mechanisms underlying protein aggregation and prionogenesis and have illuminated our understanding of human protein-folding diseases.

[*SWI*^+^] is the prion form of Swi1 [14], a subunit of the SWI/SNF chromatin-remodeling complex in yeast. Prionization of Swi1 affects the transcription of approximately 15–28% of yeast genes, even though only ~10% of yeast promoter regions are occupied by SWI/SNF [15,16,17]. Despite displaying a partial loss-of-function phenotype on non-glucose carbon source usage and a lack of multicellular features similar to the null mutant of *SWI1* [14,18], [*SWI*^+^] exhibits a distinct mRNA profile when compared with that of isogenic wild-type and *SWI1* deletion strains, suggesting a gain-of-function outcome upon adopting the Swi1 prion conformation [16,17]. The yeast Swi1 protein contains three sub-regions: the N-terminal region that is asparagine-rich (N, residues 1–323), the middle region that is glutamine-rich (Q, residues 342–524), and the C-terminal region (C, residues 525–1314) that is required for chromatin remodeling function [19]. We have previously shown that the N-region contains the prion domain (PrD) that is essential and sufficient for [*SWI*^+^] formation and propagation [20,21]. Intriguingly, a region that contains the first 32–38 amino acids of Swi1 is largely asparagine-rich, can join [*SWI*^+^] aggregates, propagate the [*SWI*^+^] conformation, and act as a transferable PrD [22,23,24]. [*SWI*^+^] relies on chaperones for its propagation and is highly sensitive to alterations in the Hsp70 chaperone system activity [14,25]. Swi1 can also interact with other prion proteins during prionogenesis and prion propagation [26,27,28,29]. Based on the tight regulation of *FLO* genes by [*SWI*^+^], a [*SWI*^+^] reporter has been developed and used in high-throughput screening for identifying anti-prion compounds [30].

For decades, tremendous efforts have been devoted to understanding the mechanisms underlying prion biology. One approach has been to modulate cellular factors/mechanisms and then examine how such modulations affect prion formation and propagation. In this line of research, molecular chaperones were found to be the major players (see recent reviews of [9,31,32,33]). The ubiquitin–proteasome [34,35] and actin cytoskeleton [36] also play important roles in prion formation and propagation. For the [*SWI*^+^] prion, we know little about what cellular factors influencing its prionogenesis and transmission. In this study, we used our established [*SWI*^+^] reporter system to identify prion destabilizers by screening the MoBY-ORF 2.0 library. The obtained candidates were further characterized for their activities against other yeast prions and analyzed for their anti-[*SWI*^+^] mechanisms.

## 2. Materials and Methods

### 2.1. Yeast Strains, Media, Plasmids, Primers, and Library

A previously created [*SWI*^+^]-containing yeast strain (LY770, BY4741 *FLO8::MET15 flo1∆::FLO1pr-URA3* [*SWI*^+^]) [18] was used as the [*SWI*^+^] strain in this study. The corresponding non-prion strain ([*swi*^−^]) was obtained by curing this [*SWI*^+^] strain with 5 mM guanidine hydrochloride. The weak, moderate, and strong [*PSI*^+^] variants used in this study were generated previously [28]. A [URE3] strain was a gift from the Wickner laboratory. The [*MOT3*^+^] strain used in this study was also described previously [30]. Extract–peptone–dextrose (YPD) media and synthetic complete (SC) media supplemented with different amino acids for auxotrophic selection were used for yeast growth. Raffinose and galactose media are SC selective media that substitute glucose with raffinose or galactose as the sole carbon source, respectively. For the raffinose media, 2 µg/anti-mycin (Sigma, St. Louis, MO, USA) was also supplemented to block the respiratory pathway, as described previously [14]. The *E. coli* DH5α strain was used as a host strain for plasmids. The plasmid *p413SWI-QC* was used to express the Swi1-QC region under the native *SWI1* promoter [18]. The plasmid *p413GAL1-NQYFP* (expressing the fusion gene of Swi1-NQ and YFP) [28] was used to evaluate the aggregation status of Swi1. The MoBY-ORF 2.0 library [37], obtained from Dr. Boone at the University of Toronto, was screened to identify [*SWI*^+^] suppressors. A *p425GAL* (ATCC, Manassas, VA, USA)-based plasmid was used as vector control. The primers used in the PCR amplification of UPTAGs and DNTAGs have been described previously [37]. The primers used in PCR to amplify 10 previously characterized anti-prion genes are listed in Table S1. The 10 anti-prion genes were PCR-amplified with PrimeSTAR DNA polymerase (TAKARA, San Jose, CA, USA) using the corresponding MoBY 2.0 plasmids as templates, and the PCR products were directionally cloned behind the *GAL1* promoter in *p425GAL1* (see Table S1 for cloning sites).

### 2.2. Primary Screen and Candidate Identification

The procedure of primary screening and candidate identification was based on a published article [37], with some modifications. Briefly, 25 µL aliquots of *E. coli* culture for each gene in MoBY 2.0 were pooled, and 0.5 mL of the mixture was used to inoculate 100 mL of LB supplemented with 100 µg/mL ampicillin and 100 µg/mL carbenicillin. After growing at room temperature for 24 h, cells were harvested, and their containing plasmids (library plasmids) were isolated using max-preparation kits (QIAGEN, Germantown, MD, USA). Competent cells of the yeast strain LY770 carrying *p413SWI-QC* were then transformed with 50 µg of library plasmids in a 5 mL solution mixture based on an LI-PEG-based protocol (Cold Spring Harbor protocol). The transformation efficiency was estimated, followed by a collection of transformants. The fresh transformants and derived cultures at 24 h, 48 h, and 72 h in SC–leu–his (extra 2% glucose was supplemented every 24 h to provide sufficient carbon source) were then spread onto SC–leu–his and SC–leu–his–ura to estimate the Ura^+^ (indicative of possible prion loss or suppression) rates. Based on the suppression results, a 3-day culture of transformants was used to screen for Ura^+^ isolates. Plasmids were then rescued from yeast cells with a QIAprep Spin Miniprep kit following a user-developed protocol (by Michael Jones, Chugai Institute for Molecular Medicine, Ibaraki, Japan, unpublished). The recovered plasmids were then used as templates for PCR amplification of the barcodes (primer pair U1-F/BupKan-R for UPTAG; BdnKan-F/D1-R for DNTAG [37]). A high-fidelity DNA polymerase (PrimeSTAR from TAKARA) was used in the PCR. The two types of PCR products were separated on a 2% LMD agarose (Sigma) gel, extracted using a protocol of freeze–squeeze [38], and purified by phenol/chloroform/isoamyl alcohol (25:24:1, Sigma). The UPTAG and DNTAG PCR products were then quantified and mixed proportionally before submission for sequencing. Based on the sequencing data, barcodes were aligned to genes in the library. Genes with more than 100 reads were picked as primary hits.

### 2.3. Prioritizing Hits and Performing Alternative Anti-[SWI^+^] Activity Test Assays

Individual plasmids of primary hits were mini-prepared from MoBY-ORF 2.0 and used to transform LY770. Individual transformants were then subjected to several rounds of assays to test their anti-[*SWI*^+^] activities. Briefly, transformants were either directly spread onto SC–leu–his and SC–leu–his–ura after proper dilution or were cultivated for 24 h before spreading to these plates. Alternatively, transformants on SC–leu–his were replica-plated onto SC–leu–his–ura to count for Ura^+^ colonies. Based on these tests, top hits were selected. Other assays were also performed to examine the prion state of the Ura^+^ isolates (aggregation and carbon source usage). The aggregation assay was conducted by a fluorescence microscopic assay [19] using LY770 cells co-transformed with a prospective suppressor plasmid and *p413GAL1-NQYFP*. A sucrose-based SC medium containing 0.05% galactose was used to induce the expression of NQ-YFP to visualize the aggregation state of Swi1. Alternative carbon source usage assays described earlier [14,18] were also conducted to confirm the prion status of Swi1.

### 2.4. Colony Visualization Assays to Test the Activities against Other Yeast Prions

As described previously [30], the prion statuses of [*PSI*^+^], [URE3], and [*MOT3*^+^] were examined based on a color change in colonies on YPD plates due to their influences on adenine biosynthesis. In all cases, prion colonies are pink or white on YPD plates, whereas non-prion colonies are red.

### 2.5. Bioinformatics Analyses

Gene lists were created at YeastMine (https://yeastmine.yeastgenome.org (accessed on 16 October 2012)). GO term enrichment and protein–protein interactions (PPIs) were conducted with Metascape [39], and the outcomes of the interaction networks were further visualized and edited by using Cytoscape 3.7.1 [40]. For enrichment assays, GO Biological Processes, KEGG Pathway, Reactome Gene Sets, and WikiPathways were analyzed (data with a *p* value < 0.01, a minimum count of 3, and an enrichment factor > 1.5 were collected). PPI analysis in this study included all 1307296 interactions recorded in the STRING, BioGrid, OmniPath, and InWeb_DB databases using a cutoff criterion (Min Network Size: 3; Max Network Size: 500). Swi1 interactors were retrieved from the Saccharomyces Genome Database (SGD, https://www.yeastgenome.org (accessed on 16 October 2021)), and *SWI1* gene regulators were collected from Yeastract (http://www.yeastract.com (accessed on 16 October 2021)).

## 3. Results

### 3.1. Eighty-Two Different Cellular Proteins Showing Suppression Activities against [SWI^+^]-Conferred Ura-Phenotype upon Overproduction

**Figure 1:**
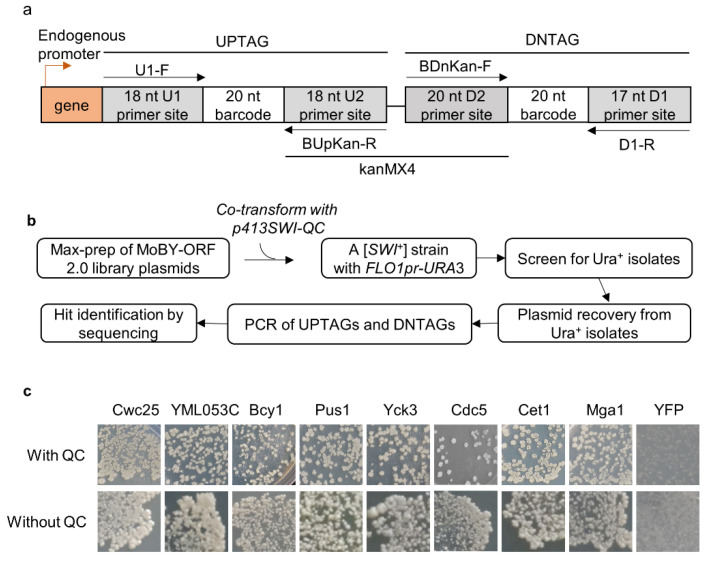
(**a**) Schematic illustration of the structure of a double barcoded yeast gene from the MoBY-ORF 2.0 library. Indicated primers (arrowed black lines) can be used to PCR-amplify the barcodes. The library was constructed with a 2 µ/*LEU2*-based plasmid vector, with each gene driven by its own native promoter (red arrow); (**b**) flowchart of the experimental procedure for screening and identification of genes that destabilize the [*SWI*^+^] prion; (**c**) the loss of [*SWI*^+^] phenotype (turning from Ura^-^ to Ura^+^) is generally independent of *p413SWI-QC*, a *CEN*-plasmid expressing Swi1-QC with the native promoter of *SWI1*, which was expressed in the tester strain LY770 to increase the sensitivity of the primary screen. Shown is the growth of the [*SWI*^+^] strain LY770 with or without Swi1-QC after 3 days of growth on SC-ura for representative prion suppressors from the MoBY-ORF 2.0 (data for more suppressors are provided in Figure S1b).

The MoBY-ORF 2.0 library carries 4527 yeast open-reading frames (ORFs) driven by their native promoters in a *LEU2*-based 2 µ plasmid [37]. In contrast to the single chromosomal copy, the library provides about 40–60 copies for each gene [41], which may boost the steady-state levels of the expressed proteins. In the library, each gene is tagged at its 3′ side with two unique barcodes of 20 nucleotides separated by the *kanMX4* gene, and there are primers available to amplify the two barcodes (Figure 1a). We speculated that we may identify some [*SWI*^+^]-curing/destabilizing ORFs under such an “overproduction” condition. LY770 is a [*SWI*^+^] strain carrying a *URA3* reporter driven by the *FLO1* promoter (*FLO1pr-URA3*) that can be used to monitor the prion state of Swi1. [*SWI*^+^] cells are Ura^-^ because the *FLO1* promoter is inactive, whereas non-prion cells ([*swi*^−^]) are Ura^+^, as functional Swi1 can activate the *FLO1* promoter [18]. In this primary assay, we also provided an extra copy of Swi1-QC (Swi1 lacking the N region) driven by the native promoter of *SWI1* (expressed from *p413SWI-QC*) with the intention of increasing the robustness of the assay. Under such a condition, Swi1-QC does not join the prion aggregates but can rather suppress the deficiency of using non-glucose carbon source of [*SWI*^+^] cells but has no detectable effect on the *FLO1pr-URA3* reporter activity [18]. As described earlier [16], approximately 7% of yeast genes are downregulated by [*SWI*^+^], and *p413SWI-QC* may help these downregulated genes to establish an overproduction condition in [*SWI*^+^] cells.

A published procedure [37], with minor modifications, was followed to identify [*SWI*^+^] destabilizer genes. Briefly, a total of 50 µg DNA of mixed library plasmids was used to transform LY770, and ~130,000 transformants (~30× of the gene number carried by the library) were collected. This large number of transformants gave us confidence that we obtained sufficient coverage of the represented genes in the library. Ura^+^ isolates were then identified. Based on what we know about [*SWI*^+^] curing by guanidine chloride that requires cell growth [25], we first tested the suppression rates by counting the resulting Ura^+^ isolates from either fresh transformants or SC cultures inoculated from fresh transformants after 1–3 days of growth. We found that allowing a period of growth dramatically increased the suppression rate, and the 3-day culture gave the highest suppression rate (Figure S1a). Thus, a 3-day culture of transformants was used in our primary screen. As illustrated in Figure 1b, after PCR amplification and sequencing the barcodes, we obtained a total of 82 hits with a read number greater than 100 (Table S2).

### 3.2. Prioritizing the Identified [SWI^+^] Inhibitors

**Figure 2:**
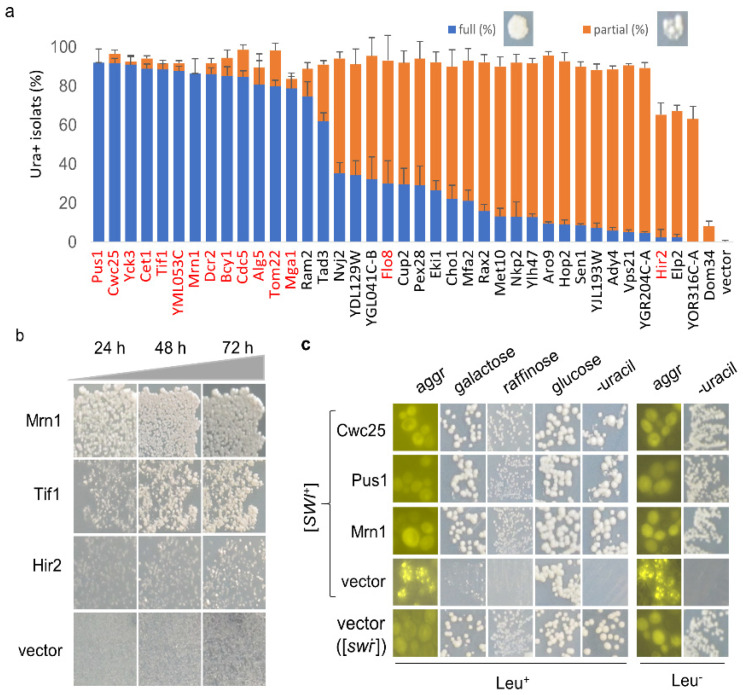
(**a**) Representative hits were quantified for their suppression efficiencies against [*SWI*^+^]. The [*SWI*^+^] strain LY770 was transformed with individual hit plasmids or vector (control) in the absence of co-expression of Swi1-QC. Transformants on SC–leu–his were replica-plated onto SC–leu–his–ura plates. After 3.5 days of growth, colonies with a full growth (full (%)) or partial growth (partial (%)) were plotted. Proteins highlighted in red were selected to be the focus of our study; (**b**) Similar to experiments shown in panel a, colonies from SC–leu–his plates were replica-plated onto SC–leu–his–ura plates that were imaged after the indicated hours of incubation. Overall, 15 hits highlighted in red in (**a**) were tested; (**c**) panels of Leu^+^: Ura^+^ isolates for the 39 hits (listed in (**a**)) obtained upon overproduction were assayed for Swi1 aggregation (aggr) and growth on SC plates with the indicated sugars as sole carbon source or on SC plates lacking uracil (-uracil). About 10-21 Ura^+^ colonies were analyzed for each hit. Panels of Leu^-^: after spontaneous loss of the overexpression plasmids, Swi1 aggregation and phenotypic suppression of the Ura^+^ isolates were examined again. Note: from panels (**a**–**c**), all strains also carried the *p413GAL1-NQYFP* plasmid, which allowed us to conduct an aggregation assay. An empty plasmid (vector) and a non-prion strain ([*swi*^−^]) were included as controls, and the representative data are shown.

Next, we extracted individual hit plasmids from the library and transformed them into LY770 independently to verify their suppression activities with co-expression of *SWI1-QC*. Three individual transformants for each hit plasmid were grown in SC–his–leu for 2.5 days before spreading onto SC selective plates lacking uracil. We found that all 82 hits could indeed suppress the Ura-phenotype of LY770 to become Ura^+^, while the vector control had no such suppression activity. Next, we tested if the expression of Swi1-QC from *p413SWI-QC* is essential for prion phenotype suppression. This was performed for 15 selected hits (indicated in red text in Figure 2a), and the results are shown in Figure 1c and Figure S1. The suppression activities were reproducible for all tested hits in the presence and absence of Swi1-QC, with only subtle differences observed for some hits. For example, in the presence of Swi1-QC, a slightly weaker suppression for Bcy1 was observed (Figure 1c). Our recent RNA-seq data suggest that Swi1-QC may reduce the expression of *BCY1*, thereby leading to slower curing kinetics. Nevertheless, the observed differences were subtle and insignificant; thus, we concluded that the suppression is *p413SWI1-QC* independent and can be assayed in the absence of the plasmid. Several rounds of subsequent tests were then performed in the absence of *p413SWI1-QC*. Among the 82 hits, we found that 38 plasmids demonstrated higher suppression effectiveness than others (Figure 2a). After replica-plating, we observed full-growth Ura^+^ colonies for 15 genes and partial-growth colonies (i.e., only part of a replicated colony was able to grow) for the rest of 23 genes (Figure 2a). Subsequent tests showed that such differences are caused by differences in suppression kinetics (Figure 2b).

### 3.3. The Nature of the [SWI^+^] Phenotypic Suppression

The conversion of LY770 from Ura^-^ to Ura^+^ may not be necessarily caused by prion loss but rather by a phenotypic mask by other mechanisms. Thus, we further investigated whether [*SWI*^+^] was indeed lost in the Ura^+^ isolates. We first carried out a fluorescence assay to examine the aggregation status of Swi1 in the converted Ura^+^ isolates upon overproduction of the hit candidates. We examined the 38 hits with higher suppressions as well as Dom34 (a weak destabilizer). Briefly, LY770 cells were co-transformed with one of the 39 plasmids and *p413GAL1-NQYFP*, which produces Swi1-NQ-YFP that forms fluorescence foci in the presence of [*SWI*^+^] but remains diffused in [*swi*^−^] cells, as described previously [19]. Upon transformation, Ura^+^ isolates were selected from SC–leu–his–ura plates and were then replica-plated onto sucrose-based SC–leu–his plates supplemented with 0.05% galactose. The obtained colonies were examined for Swi1-NQYFP aggregation. As expected, when transformed with vector Swi1 prion cells exhibited fluorescence foci, whereas [*swi*^−^] cells showed diffused signals (Figure 2c, left column). Ura^+^ isolates generated upon overproduction of the 39 hits showed no Swi1-NQ-YFP aggregation (Figure 2c). As expected, the suppression activity of Dom34 was extremely low, confirming that it was a very weak suppressor (Figure 2a). Taken together, this demonstrated that the conversion of LY770 from Ura^-^ to Ura^+^ corresponds to the loss of Swi1 aggregation.

We showed previously that [*SWI*^+^] cells are not able to use alternative carbon sources other than glucose due to Swi1 aggregation and sequestration of other transcription factors required for metabolizing alternative carbon sources, such as galactose or raffinose [14,18]. Therefore, we further examined whether the obtained Ura^+^ isolates had indeed regained the ability to use galactose upon overexpression of a suppressor gene. Specifically, we examined the hit candidates Cwc25, Pus1, and Mrn1. As shown in Figure 2c, all tested Ura^+^ isolates upon overproduction of Cwc25, Pus1, or Mrn1 were able to use galactose or raffinose as the sole carbon source—the phenotypes expected for a [*swi*^−^] strain. Under identical conditions, Ura^-^ isolates of the [*SWI*^+^] strain carrying a vector plasmid grew poorly in galactose and raffinose (Figure 2c). To further verify our results, the Leu^+^ overexpression plasmids for some hits (Cwc25, Pus1, Mrn1, Dom34) and the vector control were removed upon sequential streaking on YPD plates from the obtained Ura^+^ isolates, and the resulting isolates were assayed again. These isolates retained the Ura^+^ phenotype and exhibited diffused Swi1-NQYFP signals for the [*swi*^−^] control (Figure 2c). Combining these observations, we concluded that the Ura^+^ isolates generated by overproduction of the identified hit proteins had indeed lost [*SWI*^+^].

### 3.4. The [SWI^+^]-Specific Feature of the Identified Prion Destabilizers

**Figure 3:**
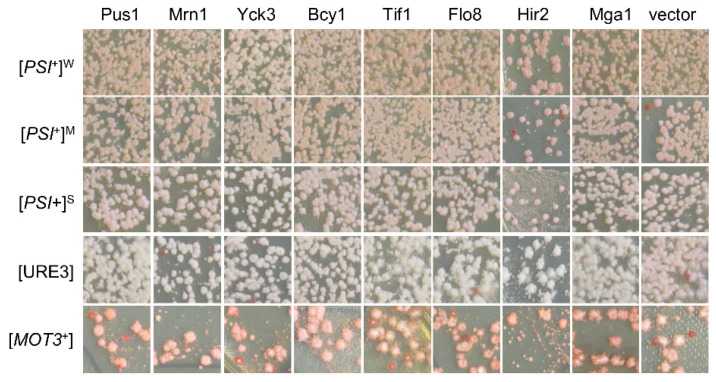
Identified [*SWI*^+^] destabilizers showed no significant effects on the propagation of the indicated non-[*SWI*^+^] yeast prions. Upon overproduction of each of the 15 proteins (in red text in Figure 2a), the statuses of the indicated prions were examined based on their colony’s colors on YPD plates, as described in the Methods. Cells losing prions would form red-like colonies on YPD. Shown are representative images from at least three repeated tests (more data are provided in Figure S1c).

We next examined the possible suppression activities of the obtained hits against three other yeast prions: [*PSI*^+^], [URE3], and [*MOT3*^+^]. This was accomplished by transforming these prions strains with the aforementioned 15 hit plasmids individually (highlighted by the red font in Figure 2a), followed by a visualization assay of their colony colors, as described in the Methods. Briefly, we specifically examined three isogenic [*PSI*^+^] variants, one [URE3] strain, and one [*MOT3*^+^] strain, all of which contain an adenine reporter, so their prion statuses can be examined together under identical experimental conditions through a visualization assay of their colony colors. All these strains were described previously [30]. In all cases, prion cells form pink or white colonies on YPD, whereas non-prion cells form red colonies. Upon prion loss (curing), a change in colony color from white/pink to red should be observed. To our surprise, there were no detectable curing effects for all 15 tested hits for the examined prions of [*PSI*^+^], [URE3], and [*MOT3*^+^] (Figure 3 and Figure S1c), suggesting that the suppression effects of the identified hits are [*SWI*^+^]-specific.

### 3.5. The Effects of a Selected Group of Documented Prion Destabilizers on [SWI^+^]

Another surprising finding of this study is that none of the previously characterized anti-prion proteins were on the list of our hit candidates. One possible explanation could be that under the examined conditions, their threshold levels for prion curing were not achieved under the regulation of their native promoters even in a high-copy number plasmid setting. To test whether this is the case, the overproduction effects of 10 documented prion-curing proteins (established upon overproduction) and several additional anti-prion proteins were examined on [*SWI*^+^] stability, including Btn2, Lug1, Nam7/Upf1, Nmd2/Upf2, Upf3, Sis1, Siw14, Ssb1, Ssz1 and Zuo1 [6,42]. Among these proteins, Sis1 was the only one tested previously for [*SWI*^+^], which cures [*SWI*^+^] when its expression is driven by a strong and constitutive promoter—*GDP* [25]. Interestingly, Sis1 was also caught in our primary screening; however, because the read number was not high enough to meet our hit criteria, it was not listed as a [*SWI*^+^]-curing hit. We sub-cloned the 10 selected ORFs into *p425GAL1* (governed by *GAL1* promoter-inducible by galactose in a 2 µ plasmid). The resulting *GAL1* plasmids and their corresponding MoBY 2.0 plasmids were used to transform LY770. The resulting transformants were then grown on sucrose-based SC–leu plates, supplemented with 2% galactose for the *GAL1* set of plasmids for overproduction. Subsequently, the colonies formed were replica-plated onto SC–leu–ura plates to test [*SWI*^+^] stability.

**Figure 4:**
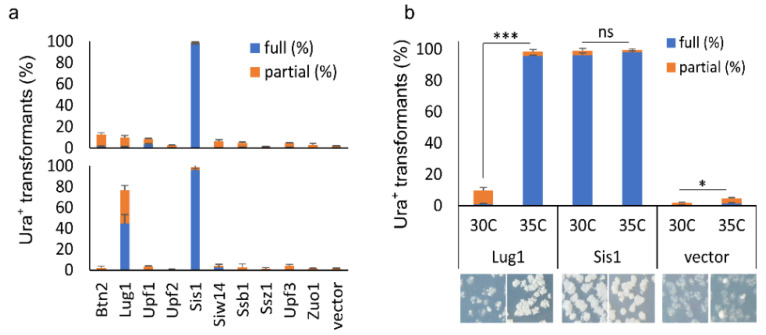
Examination of [*SWI*^+^]-curing activities of a group of documented anti-prion proteins: (**a**) all 10 indicated proteins were expressed in 2 μ plasmids, driven either by their native promoters (with plasmids from the MoBY 2.0, upper panel) or by *GAL1* promoter in a 2 μ plasmid (lower panel). Transformants of LY770 grown on SC–leu plates (for endogenous promoters) and sucrose-based SC–leu plates with 2% galactose (for *GAL1* promoter) were replica-plated onto SC–leu–ura after 3 days of growth. Transformants that fully (full (%)) and partially (partial (%)) grew on uracil-minus SC plates (Ura^+^) were then scored after 3 days of incubation; (**b**) assays were performed similarly to (**a**) except that transformants were either grown in an optimal temperature (30 °C) or in an elevated temperature (35 °C), with representative images shown at the bottom. Significance was estimated by *t*-test with the criterion—not significance (ns), *p* > 0.01; *, *p* < 0.01; ***, *p* < 0.0001. Data in this figure were from three independent transformation experiments. Vector, *p425GAL1*.

We observed that the Swi1 prion was absent from the majority of colonies transformed with either *p425GAL1-SIS1* or MoBY2.0-Sis1 plasmid, confirming the destabilizing effect of Sis1 upon overexpression (Figure 4a, upper panel). However, the other nine ORFs from the MoBY2.0 library only slightly destabilized the prion (<10% of the tested transformants lost the prion), suggesting that they are not effective [*SWI*^+^] destabilizers. Similar results were obtained with the *GAL1* set of plasmids for the other nine ORFs, except for Lug1 (Figure 4a lower panel). We found that approximately 80% of colonies transformed with *p425GAL1-LUG1* had fully or partially lost the prion upon induction, strikingly different from its MoBY 2.0 version that only slightly destabilized [*SWI*^+^] (comparing the upper and lower panels of Figure 4a). This result suggests that Lug1 can only cure/destabilize [*SWI*^+^] when it is highly overproduced, as in this case with a strong inducible *GAL1* promoter in a 2 μ plasmid. Apparently, such an overproduction level could not be achieved with its MoBY 2.0 counterpart. We next tested if the overproduction threshold level of Lug1 for [*SWI*^+^] curing could be achieved when cells are under certain stressful conditions. We incubated the [*SWI*^+^] cells harboring the *LUG1* MoBY 2.0 plasmid either at an optimal temperature (30 °C) or at a chronically elevated temperature (35 °C). Consistent with an earlier report [25], [*SWI*^+^] cells with vector control showed slight but significant destabilization of the prion at the elevated temperature (Figure 4b). The Sis1 overproduction caused prion loss in both conditions (Figure 4b). For Lug1, the elevated temperature significantly increased the prion curing efficiency, approximately 10-fold higher when compared with the same cells grown at 30 °C (Figure 4b). Taken together, our data suggest that the curing of [*SWI*^+^] by some suppressors can be caused by changes in environmental conditions.

### 3.6. Possible Mechanisms of [SWI^+^] Destabilization by Identified Suppressors

**Figure 5:**
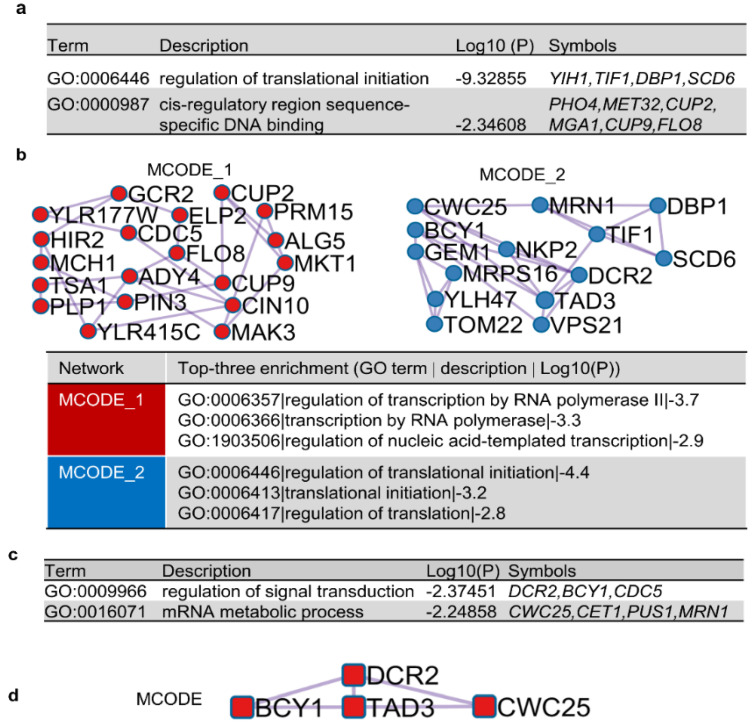
(**a**) Biological Processes (BP) enrichment of GO terms for the 82 primary hits; (**b**) protein–protein interaction (PPI) networks identified for the 82 hits. Enriched GO terms (BP) assigned for each molecular complex detection (MCODE) are shown at the bottom; (**c**) BP enrichment of GO terms for the top 15 anti-[*SWI*^+^] proteins with faster curing kinetics; (**d**) a PPI network (MCODE) was identified among the 15 top suppressors. No enrichments were identified for 23 slow [*SWI*^+^] destabilizers. Analyses in this figure were performed with Metascape (see Methods for details).

To better understand the underlying mechanisms of [*SWI*^+^] destabilization by the identified suppressors, we carried out bioinformatics analysis to identify possible pathways, functions, and cellular components targeted by those hits. We found that the 82 verified hits were involved in diverse GO terms. Although no significant enrichment was found for GO terms in specific molecular functions or cellular components, we found significant enrichment for biological processes, specifically an enrichment in two GO terms related to transcriptional regulation and translational initiation (Figure 5a). Protein–protein interaction (PPI) analyses also identified two molecular complex detection (MCODE) networks that are potentially interesting (Figure 5b). The top three GO enrichments of MCODE 1 are related to transcription, whereas the top three GO enrichments of MCODE 2 are related to translation. When a similar enrichment analysis was applied for the top 38 destabilizers only (Figure 5c,d), no enrichments were identified for the 23 [*SWI*^+^] destabilizers with slower kinetics. For the 15 prion destabilizers with faster kinetics, enrichments were found for GO terms that are associated with signal transduction and mRNA metabolic processes (Figure 5c). In the PPI analysis with the top 15 prion destabilizers with faster kinetics, only one MCODE was identified, which is composed of Bcy1, Dcr2, Tad3, and Cwc25 (Figure 5d), suggesting that these suppressors destabilize [*SWI*^+^] by targeting the same pathway with a similar mechanism. Taken together, our analyses suggest that transcription, translation, signal transduction, and mRNA metabolism were targeted by the identified [*SWI*^+^] suppressors.

Considering that downregulation of *SWI1* expression may lead to a decrease in Swi1 monomer production and thus cause prion loss, we examined whether downregulation of *SWI1* transcription was one outcome of concerted actions of the identified diverse targets. To test this possibility, 149 *SWI1* regulators were retrieved from SGD, 14 of which are *SWI1* transcriptional repressors. However, none of these *SWI1* repressors were found among the identified 82 hits, suggesting that downregulation of *SWI1* transcription was unlikely a prion curing mechanism for these identified hits. Another possible [*SWI*^+^]-curing mechanism could be a competition for Swi1 monomer between [*SWI*^+^] aggregates and Swi1-interacting proteins. In this regard, 332 *SWI1* interactors were retrieved from SGD, and 4 of them (Flo8, Elp2, Tad3, Alg5) were among the identified hits. Although there is no evidence to show such interactions are physical according to data from SGD, the overproduction of these interactors may indirectly sequester the Swi1 monomer away from the prion aggregates because of the elevated engagement of the SWI/SNF complex. As a result, the competition may limit Swi1 monomers to join [*SWI*^+^] aggregates, promoting Swi1 toward its functional pathway and thus causing prion loss.

## 4. Discussion

In this study, we identified a large number of proteins that could eliminate or destabilize [*SWI*^+^] when expressed under their native promoters from a 2 µ plasmid (Figure 2). Our results indicated that these prion inhibitors are [*SWI*^+^]-specific, as they showed no activities against several other tested yeast prions (Figure 3). We also conducted experimental and bioinformatics analyses to address their possible mechanisms of antagonizing [*SWI*^+^] (Figure 4 and Figure 5).

For decades, extensive research has been carried out to identify protein factors influencing prion initiation and propagation events. This line of research has been conducted mainly with the yeast prion [*PSI^+^*], [*RNQ*^+^], and [URE3] [9,31,32,33], and has identified a broad range of protein factors that play roles in modulating prion formation and propagation, including molecular chaperones, proteins linked to ubiquitin proteolysis, and several other anti-prion proteins [33,35,36,42]. From these studies, multiple anti-prion mechanisms have been proposed [6,43], such as facilitating prion protein folding, competing with the amyloid filaments for prion protein monomer, regulation of levels of inositol polyphosphates, and prion seed depletion caused by service cessation, asymmetric segregation, dissolution, or degradation. Importantly, these mechanisms are not mutually exclusive, and their actions may depend on specific prions and environmental conditions. These findings have greatly accelerated our understanding of prion-interacting machines and prion curing phenomena. However, very limited data are available for the [*SWI*^+^] prion, and before this study, we only knew that [*SWI*^+^] can be eliminated via Hsp104 deletion, not through its overproduction [14], and by altering the activities of the Hsp70 system chaperones or co-chaperones [25]. In this study, we identified a large number of [*SWI*^+^] suppressors. The fact that several Swi1 interactors are among the identified hits suggests that competing for Swi1 monomers between [*SWI*^+^] aggregates/seeds and overproduced Swi1 interactors may be a mechanism contributing to their [*SWI*^+^] curing. It is possible that such competition may cause a decrease or depletion in Swi1 aggregates and seeds and ultimately prion loss. Further research is needed to explore other mechanisms contributing to [*SWI*^+^] cure/destabilization. One surprising finding in this study is that the [*SWI*^+^] suppressors identified in this study could not destabilize any of the three other examined yeast prions (Figure 3). One can speculate that this may be associated with the unique features of [*SWI*^+^] and its linked alterations in SWI/SNF function. Swi1 is a low-abundance protein, and its expression is strictly regulated (SGD and [19]). In agreement with this, [*SWI*^+^] cells carry fewer prion seeds and are less stable than other well-studied prions, such as [*PSI*^+^], [URE3], and [*RNQ*^+^] [25]. Therefore, [*SWI*^+^] can be considered metastable, and a subtle balance between Swi1 prion and non-prion may be only barely maintained inside the cell. Thus, any changes (cellular and/or environmental) that break such a balance may lead to [*SWI*^+^] loss. In addition to the aforementioned competition with reported Swi1 interactors for Swi1 monomers, other events might have similar effects. For example, our bioinformatics analyses suggest that the identified [*SWI*^+^] suppressors are enriched in proteins targeting transcription, translation, signal transduction, and metabolism of mRNA and protein. Overproduction of these hit proteins may modify the activities of these pathways, leading to alterations in gene expression directly or indirectly.

Our finding that many well-studied prion suppression proteins, such as Ssb1,2, Ssz1, Zuo1, Btn2, Cur1, Hsp104, Upf1,2,3, Siw14, Sis1, and Lug1 (summarized in [6,42]), were not on the list of hit suppressors identified from our screening, was unexpected. In particular, Sis1, Ydj1, Sse1, and Sse2, which were previously shown to cure [*SWI*^+^] [25], were not among our identified hits. Our results revealed that overproduction of Lug1 and Sis1 significantly destabilized [*SWI*^+^], but the overproduction of Siw14, Btn2, Ssb1, Ssz1, Zuo1, and Upf proteins did not (Figure 4). One possible explanation for these observed differences is that our experiments were conducted under different conditions under which their threshold levels of overproduction for prion curing were not achieved. Indeed, the overproduction of many prion-curing proteins examined previously [6,25,42] was achieved by strong promoters in 2 µ plasmids. For example, the [*SWI*^+^] cure by Hsp70 chaperones or co-chaperones was observed only when expressed from a 2 µ plasmid driven by the *GPD* promoter but not by their native promoters [25]. Our finding that the curing efficiency of Lug1 is promoted by a chronically elevated heat condition under its native promoter (Figure 4b) provides additional evidence supporting such an explanation, suggesting that prion curing can be regulated by changes in environmental conditions. Our screening procedure was carried out under non-stressful experimental conditions, and the expression of MoBY-ORFs was driven by their native promoters. Thus, the overproduction levels of some of these suppressors might have not reached their curing threshold levels. In addition, the majority of identified prion antagonistic proteins were based on studies of [*PSI^+^*] and [URE3] [42]. Although common anti-prion factors indeed exist, such as the chaperone machinery of Hsp104-Hsp70s-Sis1, many of them are prion-specific [25,44,45], which might be the reason why they were not on our anti-[*SWI*^+^] candidate list. In addition, the fact that Sis1 was not identified from our primary screen but showed strong anti-[*SWI*^+^] activity when tested individually using the MoBY-Sis1 plasmid or a stronger overexpression condition (*pGAL425-SIS1*) suggests that our primary screen was not exhaustive under our specified experimental conditions. Taken together, we identified a large number of [*SWI*^+^] destabilizers, and further studies to understand their underlying mechanisms will likely provide valuable information on how prions are formed and maintained in vivo.

## Data Availability

Not applicable.

## Supplementary Materials

The following supporting information can be downloaded at https://www.mdpi.com/article/10.3390/v14071366/s1, Figure S1: Suppression features of prion destabilizing proteins, Table S1: Primers used for PCR, Table S2: List of primary hits against [*SWI^+^*].
