# Supplementary material for: Identifying Endogenous Cellular Proteins Destabilizing the Propagation of Swi1 Prion upon Overproduction
Source: Viruses. 2022 Jun 23;14(7):1366. doi: 10.3390/v14071366 (PMC9321512; doi:10.3390/v14071366)
Supplement: Supplementary file 1 [file viruses-14-01366-s001.zip › Figure S1_Suppression features of prion destablizing proteins.zip!/Figure S1_Suppression features of prion destablizing proteins.pdf]

Figure S1

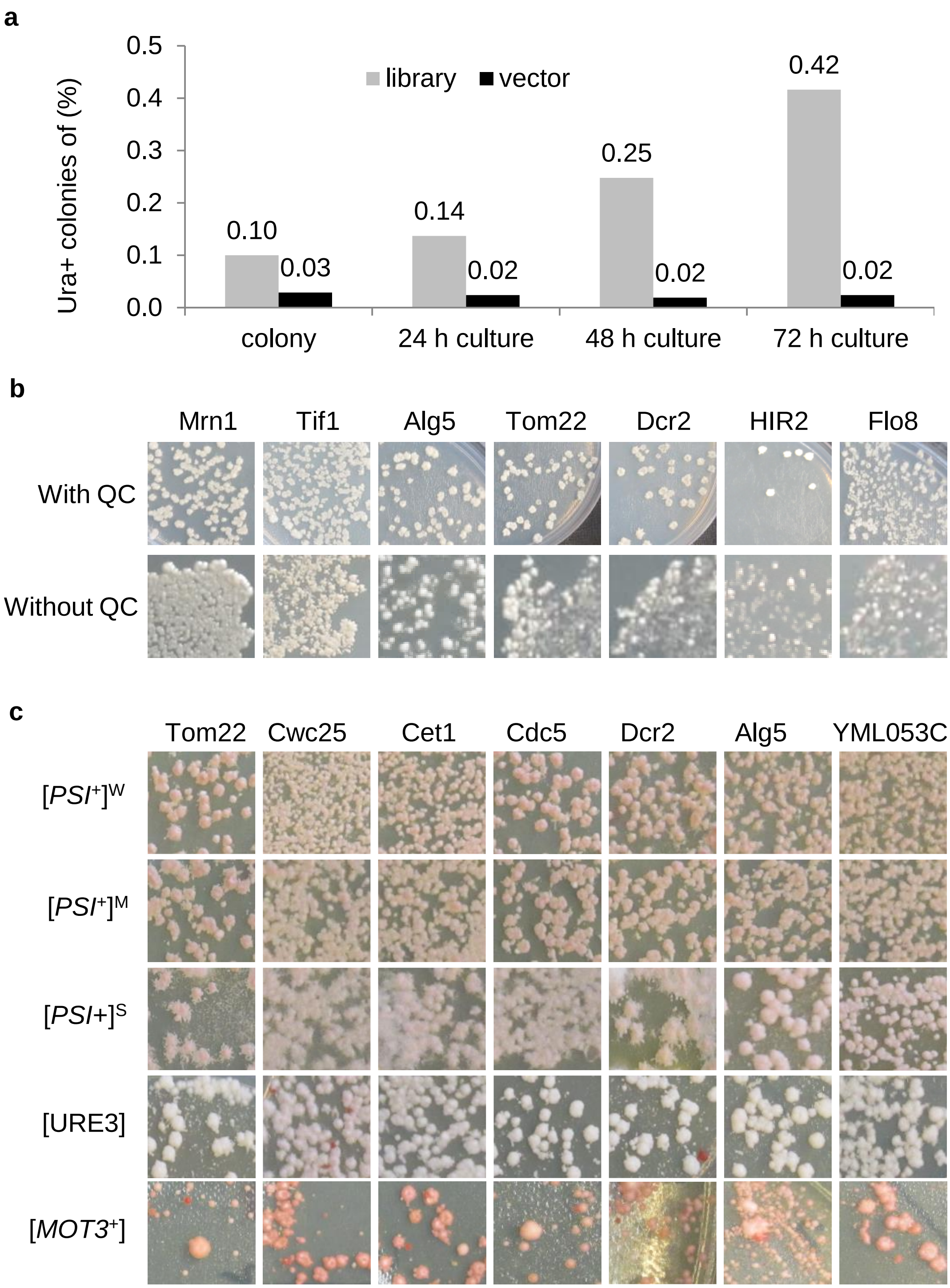

Figure S1. **(a)** Estimation of the phenotypic suppression of [*SWI*$^{+}$] upon expression of the MoBY 2.0 library. Upon co-transformation of LY770 with the library or vector with p*413SWI-QC*, fresh pooled transformants (colony) and cultures with the indicated cultivation times were spread onto to score for Ura^{+} (the suppression of [*SWI*$^{+}$] phenotype – Ura^{-}). **(b)** The Swi1-QC independency of the Ura^{-} suppression (see Figure 1c for more data). Shown are growth the tester strain with or without *p413SWI-QC* on a selective medium lacking uracil. **(c)** Anti-[*SWI*$^{+}$] hits showed no significant affects on propagation of other yeast prions (see Figure 2 for more data).
